# Reported sources of health inequities in Indigenous Peoples with chronic kidney disease: a systematic review of quantitative studies

**DOI:** 10.1186/s12889-021-11180-2

**Published:** 2021-07-23

**Authors:** Tania Huria, Suzanne G. Pitama, Lutz Beckert, Jaquelyne Hughes, Nathan Monk, Cameron Lacey, Suetonia C. Palmer

**Affiliations:** 1 Māori Indigenous Health Institute, University of Otago Christchurch, 2 Riccarton Ave, Christchurch, 8140 New Zealand; 2 Department of Medicine, University of Otago Christchurch, Christchurch, New Zealand; 3 Menzies School of Health Research, Darwin, Australia; 4 Department of Psychological Medicine, University of Otago Christchurch, Christchurch, New Zealand

## Abstract

**Background:**

To summarise the evidentiary basis related to causes of inequities in chronic kidney disease among Indigenous Peoples.

**Methods:**

We conducted a Kaupapa Māori meta-synthesis evaluating the epidemiology of chronic kidney diseases in Indigenous Peoples. Systematic searching of MEDLINE, Google Scholar, OVID Nursing, CENTRAL and Embase was conducted to 31 December 2019. Eligible studies were quantitative analyses (case series, case-control, cross-sectional or cohort study) including the following Indigenous Peoples: Māori, Aboriginal and Torres Strait Islander, Métis, First Nations Peoples of Canada, First Nations Peoples of the United States of America, Native Hawaiian and Indigenous Peoples of Taiwan. In the first cycle of coding, a descriptive synthesis of the study research aims, methods and outcomes was used to categorise findings inductively based on similarity in meaning using the David R Williams framework headings and subheadings. In the second cycle of analysis, the numbers of studies contributing to each category were summarised by frequency analysis.

Completeness of reporting related to health research involving Indigenous Peoples was evaluated using the CONSIDER checklist.

**Results:**

Four thousand three hundred seventy-two unique study reports were screened and 180 studies proved eligible. The key finding was that epidemiological investigators most frequently reported biological processes of chronic kidney disease, particularly type 2 diabetes and cardiovascular disease as the principal causes of inequities in the burden of chronic kidney disease for colonised Indigenous Peoples. Social and basic causes of unequal health including the influences of economic, political and legal structures on chronic kidney disease burden were infrequently reported or absent in existing literature.

**Conclusions:**

In this systematic review with meta-synthesis, a Kaupapa Māori methodology and the David R Williams framework was used to evaluate reported causes of health differences in chronic kidney disease in Indigenous Peoples. Current epidemiological practice is focussed on biological processes and surface causes of inequity, with limited reporting of the basic and social causes of disparities such as racism, economic and political/legal structures and socioeconomic status as sources of inequities.

## Background

The United Nations Declaration of the Rights of Indigenous Peoples asserts that Indigenous Peoples have an equal right to the highest attainable standard of physical and mental health [1]. Despite this, the health of Indigenous Peoples, particularly those who have been colonised, is unequal when compared to the health of majority populations [2]. Indigenous Peoples continue to experience health inequities in the incidence and outcomes of non-communicable diseases, including chronic kidney disease [2–4]. The health consequences of chronic kidney disease disproportionally impact Indigenous Peoples including onset at a younger age, higher rates of dialysis, lower access to kidney transplantation and premature mortality [4–6].

While a substantial literature exists to evaluate the determinants of unequal health outcomes of Indigenous Peoples and minority populations, inequities have often been explained via individual “biological risk factors”, as opposed to identifying structural and systemic perpetrators of health inequities, including racism and coloniality [6–8]. A widely accepted hypothesis to explain the inequitable burden of kidney disease in Indigenous Peoples is the higher rates of exposure to risk factors for disease including poverty, diabetes, hypertension, and cardiovascular disease and low birth weight [9, 10]. The concept of Race has been considered as equivalent to a “biological risk factor [6]. Similarly, Indigeneity has been used in research as a biological risk factor to explain health inequities associated with non-communicable diseases including chronic kidney disease [11]. The application of Indigeneity as a risk factor within statistical modelling is problematic, as it perpetuates “biological” inferiority as a primary causative factor and fails to recognise the systemic impacts of colonisation. There is a commonality of experience between Indigenous Peoples globally, and that is the ongoing impact of colonisation on health outcomes. As a consequence, epidemiological research that does not analyse the role of colonisation as a central determinant of health inequities of Indigenous Peoples will not adequately examine the root causes of inequity arising from migration, marginalisation, and racism to address inequities [7]. Indigenous health experts have called for the halt to research being done on Indigenous Peoples, and called for the adoption of research approaches in which health research agendas are led by Indigenous worldviews and researchers to increase deeper understanding of health disparities and thereby address them [7]. The ‘power of data’ to (mis) inform understandings of Indigenous health outcomes, especially the acceptance of deficit framing and reinforcement of racial profiling as causal factors for inequities, truncates opportunities to reduce disparities through policy and healthcare reform [12]. Understanding the relationship between power, colonisation, and loss of resources and the impact that these factors have on Indigenous health is a field of health research that can provide a more rigorous exploration of Indigenous health inequities to inform practice and policy [12–14].

We conducted a systematic review with quantitative analysis with the aim to summarise the reported basis related to causes of inequities in chronic kidney disease among Indigenous Peoples.

## Methods

The investigators employed a Kaupapa Māori approach to undertake this systematic review and meta-synthesis of quantitative studies evaluating chronic kidney diseases including Indigenous Peoples [15]. The Kaupapa Māori approach is an Indigenous methodology that centres Māori (Indigenous Peoples of Aotearoa New Zealand) perspectives within the research, identifies systemic barriers that maintain Indigenous health inequities and critiques colonial norms within research that silence who is being privileged (in this case within the health services). The authors TH, SGP and CL are Indigenous health researchers. LB and SCP are non-Indigenous researchers with Indigenous health research and education experience. NM is a non-Indigenous research assistant.

### Search strategy and study selection

Electronic searches of MEDLINE, Google Scholar, OVID Nursing, CENTRAL and Embase, were conducted from database inception to 31 Dec 2019 using the keywords “Indigenous”, “chronic kidney disease”, “end-stage renal disease” and “end-stage kidney disease.” After removing duplicate reports, the titles and abstracts of retrieved citations were screened according to the inclusion criteria independently by two investigators (TH and NM). TH and NM discussed abstracts requiring a consensus decision. Any differences that arose were resolved by discussion or with a third investigator (SCP or SGP). The full text of records meeting the criteria were then examined by TH.

Studies were eligible if they were an observational study design (case series, case-control, cross-sectional or cohort studies) in which the epidemiology of chronic kidney disease was evaluated in the following Indigenous Peoples who continue to experience colonisation: Māori, Aboriginal and Torres Strait Islander, Métis, First Nations Peoples of Canada, First Nations Peoples of the United States of America, Native Hawaiian and the Indigenous Peoples of Taiwan. Studies were limited to publications in the English language and studies of adults aged 18 years or older. A systematic review protocol was not registered.

### Data extraction

Data were extracted from each study into a purpose-built database by a single investigator (TH). The extracted variables were study characteristics, including populations, settings, exposures, study methods and outcomes.

### Data synthesis

Two cycles of analysis of extracted data were conducted aligned with a Kaupapa Māori methodology, to support the critique of systemic barriers and colonial norms that maintain health inequities. A Kaupapa Māori approach requires a Māori researcher to lead the study development and research process. The review was conducted utilising the David R Williams framework for studying racial differences in health to analyse reported sources of Indigenous inequities in chronic kidney disease [6]. The David R Williams framework was used to structure the first cycle of analysis to centre the exploration of racism as a basic cause of inequity. The David R Williams framework explores racial differences with the following headings (and subheadings): basic causes (*culture, biology/geographical origins, racism, economic structures, political/legal*), social status (*socioeconomic status, race, gender/age/marital status*), surface causes (*health practices, stress, psychosocial resources, medical care*), biological processes (*endocrine, metabolic, immune, cardiovascular*) and health status (*morbidity, mortality, disability, mental health, positive health*) [6]. In the first cycle of coding, a descriptive synthesis of the study research aims, methods and outcomes was used to categorise findings inductively based on similarity in meaning using the David R Williams framework headings and subheadings. In the second cycle of analysis, the numbers of studies contributing to each category were summarised by frequency analysis.

A sub-group analysis explored frequencies occurring in two distinct time periods (1994–2005 and 2006–2019), and in studies with or without reporting an Indigenous methodology for study conduct.

### Assessment of completeness of reporting

The Consolidated Criteria for strengthening reporting of health research involving Indigenous Peoples (CONSIDER) were used to evaluate the completeness of reporting. The CONSIDER statement is a checklist for reporting of research methodologies based on ethical guidance for research involving Indigenous Peoples [16].

## Results

### Baseline characteristics

**Fig. 1:**
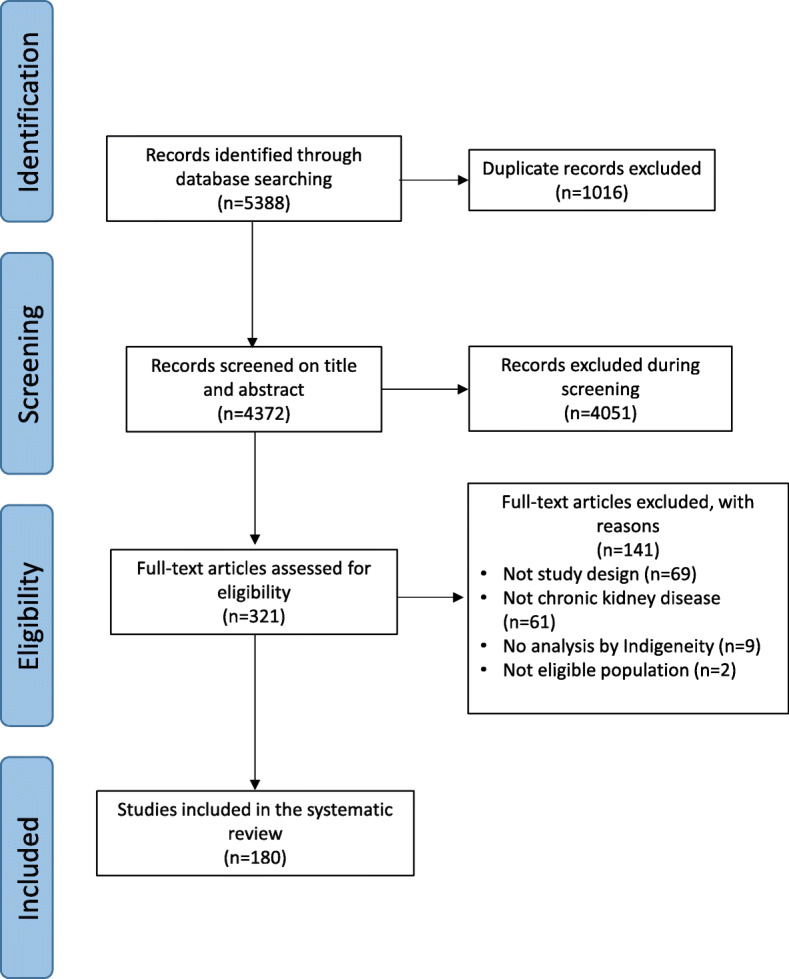
Study Identification

The search screened 4372 records (Fig. 1). Of these records 180 studies proved eligible. Over half of the eligible studies involved Aboriginal and Torres Strait Islander participants (73 studies (40%)) and First Nations Peoples of the United States of America (49 studies (27%)). (Table 1) The number of Indigenous participants in the studies ranged from 3 to 48,669. Government funding of the research was reported in 93 (52%) studies.

**Table 1:** Characteristics of included studies

Characteristics	No. (%) ***N*** = 180
**Indigenous peoples**	–
Aboriginal and Torres Strait Islanders (Australia)	73 (40)
Metis and First Nations (Canada)	35 (19)
Native Hawaiian (Hawaiian Islands, United States of America)	1 (1)
Māori (Aotearoa)	21 (12)
Taiwanese Indigenous Peoples (Taiwan)	1 (1)
First Nations Peoples (United States of America)	49 (27)
**Indigenous research participation**	–
Methodology	7 (4)
Consultation/Advisory/Ethics	49 (27)
Funding	8 (4)
None of the above stated	116 (64)
**Source of funding**	–
Government	93 (52)
Non-Government Organisation	15 (8)
Industry	6 (3)
Indigenous	8 (4)
Not stated	58 (32)
**Research setting**	–
Database/registry	60 (33)
Hospital/outpatient	22 (12)
Primary care	25 (14)
Community	34 (19)
Dialysis unit	23 (13)
Genetic samples/laboratory samples	16 (9)
**Year of publication**	–
1990–1995	1 (1)
1996–2000	2 (1)
2001–2005	7 (4)
2006–2010	66 (37)
2011–2015	70 (39)
2016–2017	22 (12)
2018–2019	12 (7)

The analyses were conducted within an existing database or registry (60 studies (33%)), community-based research (34 studies (19%)), primary care or rural clinics (25 studies (15%)), dialysis and transplant units (23 studies (13%)), hospital/outpatient (22 studies (12%)) and analysis of collected laboratory or genetic samples (16 studies (9%)).

### Completeness of reporting

Based on CONSIDER reporting checklist, research governance was reported in two studies research prioritisation based on Indigenous stakeholder perspectives or empirical data was not reported in any study and the methodological approach to the inclusion of Indigenous participants was reported in 34 studies. (Table 2) Of the eligible studies 12 did not report current or future consent for tissue storage.

**Table 2:** Completeness of reporting using the CONSIDER checklist

Item	Checklist Item	No. of studies reporting number (%)	References
**Research Governance (Indigenous governance and Research Institutions)**			
1	Describe partnership agreements between the research institution and Indigenous-governing organization.	2 (1)	[17, 18]
2	Describe any accountability/review mechanism within the partnership agreement that addresses harm minimization.	0	–
3	Specify how the research partnership agreement includes the protection of Indigenous intellectual property and knowledge arising from the research, including financial and intellectual benefits generated.	0	–
**Research Prioritization**		–	–
4	Explain how the research aims emerged from priorities identified by either Indigenous stakeholders, empirical evidence.	0	–
**Research Relationships (Indigenous stakeholders/participants and Research team)**		–	–
**5**	Specify measures that adhere to and honour Indigenous ethical guidelines.	8 (4)	[16, 17, 20–25]
6	Report how Indigenous stakeholders were involved in the research processes (i.e., research design, funding,	42 (23)	[17, 20–22, 26–64]
7	Describe the expertise in Indigenous health and research of the research team.	2 (1)	[16, 65]
**Research Methodologies**		–	–
8	Describe the methodological approach of the research, including a rationale of methods.	6 (3)	[16, 47, 66–69]
9	Describe how the research methodology incorporated consideration of the physical, social, economic, and cultural environment of the participants and prospective participants.	34 (18) (22)	[3, 16, 17, 21–25, 27–33, 42, 43, 45, 47, 51, 52, 56–58, 62, 66, 68, 70–76]
**Research Participation**		–	–
10	Specify how individual and collective consent was sought to conduct future analysis on collected samples and data, other than what was the approved initially (e.g., third parties accessing samples (genetic, tissue, blood) for additional analyses).	0	–
11	Provide details on how the resource demands (current and future)	0	–
12	Specify how biological tissue and other samples, including data, were stored/disposed of.	0	–
**Research Capacity**		–	–
13	Explain how the research supported the development and maintenance of Indigenous research capacity	11 (6)	[20, 27, 33, 37, 39, 40, 47, 74, 78, 77, 79]
14	Discuss how the research team undertook professional development opportunities	0	–
**Research Analysis and Interpretation**		–	–
15	Specify how the research analysis and reporting supported critical inquiry and a strength-based approach.	0	–
**Research Dissemination**		–	–
16	Describe how the research findings were disseminated to relevant Indigenous governing bodies and peoples.	0	
17	Discuss the process for knowledge translation and implementation to support Indigenous advancement	0	

### Analysis of health differences

#### Basic causes

**Fig. 2:**
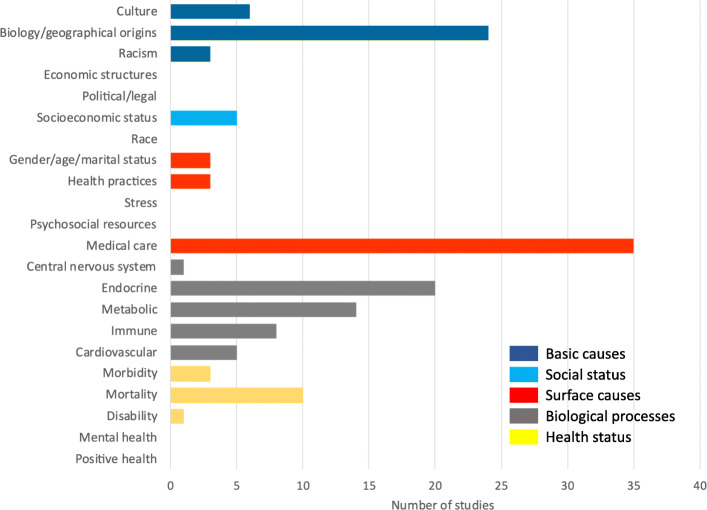
Summary of reported sources of health inequities in Indigenous Peoples with chronic kidney disease using the David R Williams framework for studying of racial differences in health

In the David R Williams framework, basic causes of health differences include culture, biology/geographical origins, racism and political or legal factors. 57 (32%) studies investigated culture (*n* = 9), biology or geographical origins (*n* = 43) or racism (*n* = 5) as causes of health inequity for Indigenous participants (Fig. 2). Economic structures and political or legal causes were not reported as a source of inequity. Of the 43 studies citing biology/geographical origins, 5 reports (3%) were based on samples taken from a single Indigenous community. Of the 9 studies (5%) with a genetic focus, genes associated with kidney function, the heritability of serum sodium concentration, genome scanning and the association of genetic markers with the risk of developing kidney failure due to diabetes were evaluated.

Investigator teams interpreted the findings of their research to indicate that colonisation and racism were determinants of chronic kidney disease risk and outcomes in Indigenous Peoples in 46 (26%) studies. In four studies, it was identified that increased understanding of culturally-specific beliefs and practices such as the return of tissue or the handling of samples led to changes in clinical practice. The investigators of these studies reported that increased understanding of cultural practices led to improved acceptability of treatment to Indigenous Peoples. The investigators also reported the need for non-Indigenous health professionals to increase cultural competencies when working with Indigenous Peoples [17, 27, 66, 70].

### Social status

Social status includes socio-economic status, race, gender, age and marital status. In 27 studies (15%), researchers identified socio-economic factors as determinants of patient outcomes related to chronic kidney disease in Indigenous patients. Reported factors included rurality and socioeconomic status. In eight of these studies, investigators reported that poverty and access to quality health services were factors associated with health outcomes for Indigenous Peoples with chronic kidney disease. No studies evaluated the intersection between race, social status and chronic kidney disease epidemiology.

#### Surface causes

Surface causes include individual health-related behaviours such as smoking and alcohol use, stress, psychosocial resources and medical care. Medical care in the David R Williams framework refers to the health system, physician care and clinical care practices such as transplantation and dialysis. Medical care was the focus of 68 studies (38%) including studies investigating transplantation and dialysis treatment patterns. Six studies all published since 2014, reported clinical care as a source of inequities in chronic kidney disease. These studies also reported improved health outcomes in Indigenous patients following clinical interventions tailored to prevent chronic kidney disease through point of contact screening in rural locations. In 21 (12%) studies, investigators reported on Indigenous patient experiences. These studies reported that Indigenous patients’ education and relationships with health providers were important aspects of their care. The studies also highlighted patient perceptions of unequal treatment, clinician bias and barriers to care such as geographical accessibility. Investigators in two studies explored screening for chronic kidney disease in primary care. Treatment pathways for Indigenous patients were investigated in 8 (4%) studies. These studies reported ways to address accessibility barriers such as point of contact screening, early prevention and education about diabetes and kidney disease, and clinical practice screening solutions [80].

Sixty-three studies (41%) reported on personal health behaviours including smoking, alcohol use and modifiable lifestyle factors including diet as contributing factors to inequities in chronic kidney disease in Indigenous Peoples.

### Biological processes

Biological processes include central nervous, endocrine, metabolic, immune and cardiovascular systems. Biological processes were the most frequently cited determinants of chronic kidney disease outcomes in the eligible studies. Investigators in 91 (51%) of the eligible studies concluded that Indigenous Peoples had increased risks of chronic kidney disease and kidney failure compared to non-Indigenous participants and that Indigenous Peoples had a higher prevalence of identifiable risk factors, such as type 2 diabetes and cardiovascular disease. Investigation of biological processes within the eligible studies was predominantly focused on endocrine (35 studies) and metabolic (30 studies) processes associated with chronic kidney disease, particularly hypertension and type 2 diabetes.

### Health status

Health status in the framework refers to morbidity, mortality, prognosis, incidence, disability, mental health and wellbeing. Investigators of included studies analysed mortality and hospitalisations in Indigenous Peoples with chronic kidney disease in 22 (12%) studies. Investigators in 18 (10%) studies evaluated differences in incidence and prevalence of chronic kidney disease in Indigenous Peoples in comparison with non-Indigenous peoples. All of these studies identified a higher burden of chronic kidney disease when compared with a non-Indigenous cohort.

### Subgroup analysis

A subgroup analysis by publication date identified that prior to 2004 there were only a small number of studies published investigating kidney disease in Indigenous Peoples (*n* = 7) [10, 11, 28, 29, 81–84]. The subgroup analysis identified that studies reporting Indigenous methodologies and research principles were more likely to have been published more recently (2012–2019) [17, 27, 66, 67, 71, 78, 85]. Investigators of studies that reported using Indigenous research principles and Indigenous methodologies described active participation and relationships with Indigenous stakeholders in research conduct. Investigator teams described components of strength-based Indigenous methodology within the analysis and interpretation of the findings, including an Indigenous worldview and the utilisation of Indigenous quantitative research methodologies, e.g., the use of the Indigenous cohort as the reference cohort. The investigators identified racism, colonisation, and social and economic disparities as causative factors of inequities related to chronic kidney disease [27, 66, 67, 78].

## Discussion

We used the meta-synthesis of epidemiological studies approach incorporating Indigenous methodologies to explore the reported sources of health inequities in Indigenous Peoples with or at risk or chronic kidney disease. The analysis employed a Kaupapa Māori approach using the David R Williams framework for studying racial differences in health. The key finding was that epidemiological investigators most frequently reported biological processes of chronic kidney disease, particularly type 2 diabetes and cardiovascular disease as the principal causes of inequities in the burden of chronic kidney disease for colonised Indigenous Peoples. Social and basic causes of unequal health including the influences of economic, political and legal structures on chronic kidney disease burden were infrequently reported or absent in existing literature.

This systematic analysis raises the possibility that a research focus on biological process and surface causes has provided a foundation of understanding of potential causative factors of inequities in chronic kidney disease experienced by Indigenous Peoples. This may have resulted in a more restricted understanding of the roles of political and economic domains as root causes of inequities for Indigenous Peoples with chronic kidney disease, limiting effective policy and practice responses [8, 12, 79]. The inclusion of broader determinants of health within epidemiological analyses, such as racism, colonisation, bias, and Indigenous perspectives may be crucial to gain a more functional understanding of inequity, to underpin effective interventions that can address Indigenous health inequities [2, 7, 86–89]. This is important as the relationship between basic causes of racial differences and the link with health status highlights power and access to resources as causative processes that lead to health inequities [8]. Researchers have the opportunity to increase understanding of exposure to risk factors of chronic kidney disease by incorporating Indigenous viewpoints and research that is responsive to Indigenous health advancement to expand exploration of socio-political causes of inequity, and thereby design health systems and practices to counter them [7, 16].

This review identified that there is still a need for greater understanding of the impact of political and social structures on Indigenous health inequities, and requires research partnerships that are enabled to consider, and explore a wider range of causes of inequity in non-communicable diseases. A strength-based process could assist to expand the influence of research from a focus on immediate biological factors, to research that is inclusive of, and driven by, Indigenous understanding, knowledge and experiences that considers racism social justice and wider socio-political factors, to inform policy and practices to address health inequities. The recognition of the impact of power on Indigenous health outcomes ultimately leads to research that is inclusive of Indigenous knowledge [12, 15, 90] by directing the focus beyond biological, genetic and race factors to address broader and modifiable sources of Indigenous health inequities including political and economic health policy [8, 91–93].

This review identified that between 2012 and 2019 there was an increase in studies incorporating Indigenous research methodologies, principles and Indigenous led research. It is plausible that the increase in Indigenous stakeholder involvement directly corelates to the higher number of research protocols/guidelines that identify the need for partnership with Indigenous stakeholders [16, 94–97]. However there is limited evidence that researchers and research funding organisations are held accountable to deliver on the promise of increased Indigenous involvement. The global spotlight seems to focus on explicit bias and racism against minority groups. Bias within competitive research funding, and publication of health research that involves Indigenous communities that is inclusive of Indigenous knowledges may not be focussed on [98, 99]. Health research needs to identify bias within its structures, and demand that research no longer perpetuates stereotypes of Indigenous Peoples with chronic conditions.

The strengths of this study include the systematic meta-analytical approach, application of David R Williams framework to the synthesis of eligible studies, the use of Indigenous research-reporting criteria (CONSIDER) to assess the completeness of reporting and utilisation of a Kaupapa Māori methodology. There are limitations of this review that need to be considered when interpreting the findings. The inclusion criteria for the studies were limited to research inclusive of Indigenous Peoples of Aotearoa New Zealand, Australia, Canada, Taiwan, Hawaii, the United States of America, and experiences of Indigenous Peoples from outside these nations have not been included. Eligible studies were limited to the English language. The David R Williams framework was initially developed to investigate differences in health based on race, rather than Indigeneity, and may not be fully applicable to the elements of coloniality that are specific to Indigenous health outcomes.

## Conclusions

In this systematic review with meta-synthesis, a Kaupapa Māori methodology and the David R Williams framework was used to evaluate reported causes of health differences in chronic kidney disease in Indigenous Peoples. Current epidemiological practice is focussed on biological processes and surface causes of inequity, with limited reporting of the basic and social causes of disparities such as racism, economic and political/legal structures and socioeconomic status as sources of inequities.

## Data Availability

Data sharing is not applicable to this article as no datasets were generated or analysed during the current study.

## Abbreviation

CONSIDER: Consolidated criteria for strengthening reporting of health research involving Indigenous Peoples
